# Novel, acidic, and cold-adapted glycoside hydrolase family 8 endo-β-1,4-glucanase from an Antarctic lichen-associated bacterium, *Lichenicola cladoniae* PAMC 26568

**DOI:** 10.3389/fmicb.2022.935497

**Published:** 2022-07-14

**Authors:** Do Young Kim, Jonghoon Kim, Yung Mi Lee, Soo Min Byeon, Jeong Hae Gwak, Jong Suk Lee, Dong-Ha Shin, Ho-Yong Park

**Affiliations:** ^1^Industrial Bio-Materials Research Center, Korea Research Institute of Bioscience and Biotechnology, Daejeon, South Korea; ^2^Division of Life Sciences, Korea Polar Research Institute, Incheon, South Korea; ^3^Department of Biological Science, Daejeon University, Daejeon, South Korea; ^4^Biocenter, Gyeonggido Business and Science Accelerator (GBSA), Suwon, South Korea; ^5^Insect Biotech Co. Ltd., Daejeon, South Korea

**Keywords:** endo-β-1, 4-glucanase, glycoside hydrolase, GH8, cold-adapted enzyme, Antarctica, lichen-associated bacterium, *Lichenicola cladoniae*

## Abstract

Endo-β-1,4-glucanase is a crucial glycoside hydrolase (GH) involved in the decomposition of cellulosic materials. In this study, to discover a novel cold-adapted β-1,4-D-glucan-degrading enzyme, the gene coding for an extracellular endo-β-1,4-glucanase (GluL) from *Lichenicola cladoniae* PAMC 26568, an Antarctic lichen (*Cladonia borealis*)-associated bacterium, was identified and recombinantly expressed in *Escherichia coli* BL21. The GluL gene (1044-bp) encoded a non-modular polypeptide consisting of a single catalytic GH8 domain, which shared the highest sequence identity of 55% with that of an uncharacterized protein from *Gluconacetobacter takamatsuzukensis* (WP_182950054). The recombinant endo-β-1,4-glucanase (rGluL: 38.0 kDa) most efficiently degraded sodium carboxymethylcellulose (CMC) at pH 4.0 and 45°C, and showed approximately 23% of its maximum degradation activity even at 3°C. The biocatalytic activity of rGluL was noticeably enhanced by >1.3-fold in the presence of 1 mM Mn^2+^ or NaCl at concentrations between 0.1 and 0.5 M, whereas the enzyme was considerably downregulated by 1 mM Hg^2+^ and Fe^2+^ together with 5 mM *N*-bromosuccinimide and 0.5% sodium dodecyl sulfate. rGluL is a true endo-β-1,4-glucanase, which could preferentially decompose D-cellooligosaccharides consisting of 3 to 6 D-glucose, CMC, and barley β-glucan, without other additional glycoside hydrolase activities. The specific activity (15.1 U mg^–1^) and *k*_cat_/*K*_*m*_ value (6.35 mg^–1^ s^–1^mL) of rGluL toward barley β-glucan were approximately 1.8- and 2.2-fold higher, respectively, compared to its specific activity (8.3 U mg^–1^) and *k*_cat_/*K*_*m*_ value (2.83 mg^–1^ s^–1^mL) toward CMC. The enzymatic hydrolysis of CMC, D-cellotetraose, and D-cellohexaose yielded primarily D-cellobiose, accompanied by D-glucose, D-cellotriose, and D-cellotetraose. However, the cleavage of D-cellopentaose by rGluL resulted in the production of only D-cellobiose and D-cellotriose. The findings of the present study imply that rGluL is a novel, acidic, and cold-adapted GH8 endo-β-1,4-glucanase with high specific activity, which can be exploited as a promising candidate in low-temperature processes including textile and food processes.

## Introduction

Cellulose, which is one of the recalcitrant polysaccharides present in nature, consists of D-glucose repeating units linked by β-1,4-glycosidic bonds in the backbone. In plant cell walls, the structural polysaccharide composed of highly ordered β-1,4-D-glucan chains is the principal constituent and is interlinked with hemicelluloses and lignin in a complex network (Horn et al., 2012). Therefore, to efficiently decompose rigid polymer networks, many cellulolytic bacterial and fungal strains in ecosystems generally produce different glycoside hydrolase (GH) enzymes including endo-β-1,4-glucanase (EC 3.2.1.4), cellobiohydrolase (EC 3.2.1.91), and β-glucosidase (EC 3.2.1.21) that are synergistically involved in the breakdown of cellulose fibrils (Dimarogona et al., 2013; Bhardwaj et al., 2021).

So far, a wide variety of cellulose-degrading mesophilic and extremophilic microorganisms have been identified from distinct ecosystems, such as soil (López-Mondéjar et al., 2016), compost (Lv and Yu, 2013), fresh and sea water (de Menezes et al., 2008; Arneen et al., 2014), the guts of animals (Dantur et al., 2015; Handique et al., 2017), Arctic and Antarctic samples (Duncan et al., 2008; Zhao et al., 2019), hot springs (Miroshnichenko et al., 2008), deep seas (Zeng et al., 2006), and soda lakes (van Solingen et al., 2001), by the isolation of pure cultures and metagenomic analysis of environmental samples (Wilson, 2011; Santiago et al., 2016; Liu et al., 2021). Accordingly, the structural and biocatalytic characteristics of endo-β-1,4-glucanases, which play a critical role in cellulose degradation, have been well studied compared to exo-type cellulases (Juturu and Wu, 2014). Endo-β-1,4-glucanases are currently categorized into six retaining GH families (5, 7, 10, 12, 44, and 51) and seven inverting GH families (6, 8, 9, 45, 48, 74, and 124) based on the amino acid sequence similarities of their catalytic domain.^1^ It has also been shown that compared to retaining endo-β-1,4-glucanases with a (β/α)_8_-barrel fold belonging to GH families 5, 10, 44, and 51, retaining endo-β-1,4-glucanases belonging to GH families 7 and 12 contain a β-jelly roll fold, although these enzymes have two catalytic glutamate (Glu) residues in common in their active site. However, unlike inverting GH6 endo-β-1,4-glucanases with a (β/α)_8_-barrel fold and two active site Asp residues, inverting endo-β-1,4-glucanases with an (α/α)_6_-barrel fold belonging to GH families 8, 9, and 48 possess highly conserved catalytic residues Asp (nucleophile/base) and Glu (proton donor) in their active site. Besides the lysozyme fold found in inverting GH124 endo-β-1,4-glucanases, inverting GH45 and GH74 endo-β-1,4-glucanases consist of a 6-stranded β-barrel fold and a 7-bladed β-propeller fold, respectively, together with two catalytic Asp residues in their active site (see text footnote 1).

Cold-adapted or cold-active enzymes with outstanding biocatalytic properties at low temperatures below 25°C have drawn a great deal of industrial attention because they are a fascinating option without the need for heating processes, which hinder the sustainability, quality, and cost-effectiveness of biotechnological production (Santiago et al., 2016). Thus, from the viewpoint of industrial biocatalysts, highly active endo-β-1,4-glucanases have also attracted a lot of interest as potential candidates for green chemistry that can be applicable in laundry detergents, food and feedstock, bio-fuel, the bio-polishing of textiles, paper-pulp processing, and pharmaceutical industries (Vester et al., 2014; Al-Ghanayem and Joseph, 2020). Some cold-adapted or cold-active endo-β-1,4-glucanases with distinct molecular structures and enzymatic properties have been identified from different organisms in extremely cold environments including the Arctic and Antarctic regions and deep-sea sediment (Yang and Dang, 2011; Song et al., 2017; Zhao et al., 2019). However, no report concerning the genetic and functional characteristics of Arctic and Antarctic microbial GH8 endo-β-1,4-glucanases active at low temperatures has been published to date, although three cold-adapted or cold-active GH8 endo-β-1,4-glucanases identified from plant-associated bacteria (Chen et al., 2020; de Melo et al., 2021) and cold desert soil in Ladakh (Bhat et al., 2013) were recently characterized at the molecular level. Therefore, to discover such GH8 endo-β-1,4-glucanases from polar microbes, we performed an *in silico* analysis of the whole genome sequence of a psychrophilic bacterium, *Lichenicola cladoniae* PAMC 26568 that was isolated from a lichen specimen of *Cladonia borealis* taken at King George Island, Antarctica (Noh et al., 2020). Herein, we report the discovery and genetic and biocatalytic characteristics of a novel, acidic, and cold-adapted GH8 endo-β-1,4-glucanase from cellulolytic *L. cladoniae* PAMC 26568.

## Materials and methods

### Materials

D-Cellooligosaccharides [D-cellobiose (C_2_) to D-cellohexaose (C_6_)], β-glucan from barley (low viscosity), curdlan from *Alcaligenes faecalis*, xyloglucan, and glucomannan were purchased from Megazyme International Ireland Ltd. (Wicklow, Ireland). All other materials including D-glucose (C_1_), sodium carboxymethylcellulose (CMC), Avicel PH-101, chitosan, beechwood xylan, locust bean gum, *para*-nitrophenyl (*p*NP)-glucopyranoside, and *p*NP-cellobioside were provided by Sigma-Aldrich (St. Louis, MO, United States).

### Molecular cloning of the endo-β-1,4-glucanase gene

Genomic DNA of *L. cladoniae* PAMC 26568 was prepared from the cells, which were cultivated in R2A broth (BD Difco, Franklin Lakes, NJ, United States) for 14 days at 15°C, using a Mini Tissue DNA kit (Cosmo Genetech Co. Ltd., Seoul, South Korea) and was used as a template in polymerase chain reaction (PCR). With a PCR thermal cycler (TaKaRa, Kyoto, Japan), amplification of the gene encoding recombinant GluL proteins (rGluL) without a signal sequence was performed using the designed two gene-specific primers GluL-F (5′-*CATATG*GATGCCGATGTCCAGTGGC-3′) and GluL-R (5′-*AAGCTT*TCATGGCGAATGTCTTTCCGTCC-3′), which had restriction sites *Nde*I and *Hin*dIII, respectively. In this case, the PCR mixture (50 μL) included 2.5 U of FastStart Taq DNA polymerase (Roche, Basel, Switzerland), 250 μM of each dNTP, 2 pmol of each PCR primer, 20 ng of template DNA, and a PCR buffer. The initial template denaturation was carried out for 4 min at 95°C, followed by 35 cycles of 30 s at 95°C, 30 s at 54.5°C, and 1 min at 72°C. The amplified PCR products were separated by electrophoresis on a 1.2% agarose gel, followed by purifying the desired gene fragments using a NucleoSpin Gel and PCR Clean-up (Macherey-Nagel, Düren, Germany). The obtained gene fragments (975-bp) were then ligated into a pGEM-T easy vector (Promega, Madison, WI, United States). After transformation of the constructed pGEM-T easy/*gluLR* vectors into *Escherichia coli* DH5α competent cells, they were purified from the recombinant cells cultured in 100 mL of ampicillin (100 mg/L)-containing Luria-Bertani (LB) broth (BD Difco, Franklin Lakes, NJ, United States) for 14 h at 37°C. Subsequently, the recombinant plasmids were digested with restriction enzymes *Nde*I and *Hin*dIII and the resulting *gluL* fragments with the corresponding sticky ends were purified employing a NucleoSpin Gel and PCR Clean-up (Macherey-Nagel). For the recombinant production of GluL proteins, the obtained gene fragments were inserted into a pET-28a(+) expression vector (Novagen, Darmstadt, Germany) with the same sticky ends, after which the constructed pET-28a(+)/*gluL* vectors were transformed into *E. coli* BL21.

### Production and purification of recombinant endo-β-1,4-glucanase proteins

To produce N-terminal (His)_6_-tagged rGluL, the cultivation of recombinant *E. coli* BL21 cells containing pET-28a(+)/*gluL* was carried out in a 5-L baffled flask, which included LB broth (1 L) and kanamycin (25 mg/L), in a rotary shaker (150 rpm) for 15 h at 30°C. The overexpression of *gluL* was induced by adding 1.0 mM isopropyl β-D-1-thiogalactopyranoside (IPTG) after the optical density of the bacterial culture at 600 nm reached around 0.45. The rGluL-expressing cells were recovered from the culture broth by centrifugation (8,000 × *g*) for 20 min at 4°C and then stored at –20°C for 3 h. For the isolation of active rGluL proteins, the frozen recombinant *E. coli* BL21 cells were thoroughly suspended in a binding buffer (pH 7.4) consisting of 20 mM sodium phosphate, 0.5 M NaCl, and 20 mM imidazole, after which they were disrupted by sonication. The soluble fraction including rGluL proteins displaying endo-β-1,4-glucanase activity toward CMC was carefully collected by centrifugation (15,000 × *g*) for 20 min at 4°C. In this study, the simple purification of rGluL proteins was achieved by affinity chromatography using a HisTrap HP (Cytiva, Uppsala, Sweden) (5.0 mL) column connected to a fast protein liquid chromatography system (Amersham Pharmacia Biotech, Uppsala, Sweden), according to the manufacturer’s protocol. The N-terminal (His)_6_-tagged rGluL proteins were collected from the column by a linear gradient elution method with imidazole (20–500 mM) at a flow rate of 2.0 mL/min. The fractions exhibiting high endo-β-1,4-glucanase activity were then collected, combined, and desalted with a HiPrep 26/10 desalting column (GE Healthcare) using 50 mM sodium phosphate buffer (pH 6.0) as the mobile phase. The desalted active fractions were recovered, pooled, and applied for further analysis.

### Protein analysis

The relative molecular mass of the denatured rGluL proteins was analyzed by sodium dodecyl sulfate-polyacrylamide gel electrophoresis (SDS-PAGE) in a 12.0% gel. After the electrophoresis, the gel was stained for 3 h with a 0.05% Coomassie Brilliant Blue R-250 solution (Bio-Rad Laboratories, Inc., Seoul, South Korea) to visualize the proteins separated by SDS-PAGE. Quantitative determination of the protein concentrations was performed using Bio-Rad Protein Assay Dye Reagent Concentrate (Bio-Rad Laboratories, Inc., Seoul, South Korea) using bovine serum albumin as a standard.

### Enzyme assays

The quantitative assay of endo-β-1,4-glucanase activity was conducted by measuring the amount of reducing sugars produced after the enzymatic degradation of CMC employing 3,5-dinitrosalicylic acid (DNS) reagent. In this case, a standard curve for the target reducing sugar by plotting the mean absorbance against the D-glucose concentration was constructed and used for the quantification of reducing sugars. The standard assay mixture (0.5 mL) was comprised of 1.0% CMC and rGluL solution (0.05 mL) diluted in 50 mM sodium citrate buffer (pH 4.0). The enzyme reactions were routinely conducted at 45°C for 10 min. After termination of the biocatalytic reaction, 0.75 mL of the DNS reagent was added to the assay mixture and then boiled for 5 min to develop the red-brown color. The absorbance of the red-brown color developed was measured at 540 nm. One unit (U) of endo-β-1,4-glucanase activity for CMC or barley β-glucan was defined as the amount of rGluL required to release 1 μmol of reducing sugar per min under standard reaction conditions.

### Effects of pH, temperature, and chemical compounds on the recombinant endo-β-1,4-glucanase activity

The effect of pH on the endo-β-1,4-glucanase activity of rGluL toward CMC was investigated at different pH values (3.0–8.5) at 45°C for 10 min using the following buffer systems at 50 mM: sodium citrate (pH 3.0–5.5), sodium phosphate (pH 5.5–7.5), and Tris-HCl (pH 7.5–8.5). However, the pH stability of rGluL in the aforementioned pH buffers was evaluated by measuring its residual endo-β-1,4-glucanase activity after stopping the enzyme reaction that was performed at 45°C for 10 min. In this case, the biocatalytic reaction was initiated by adding 1.0% CMC to the reaction mixture after pre-incubation of 1 h at 3°C in the absence of substrate. The optimum temperature of rGluL to degrade CMC was examined by reacting it with substrate at 3, 10, 18, 25, 30, 35, 40, 45, 50, 55, 60, and 65°C for 10 min in 50 mM sodium citrate buffer (pH 4.0). The thermal stability of the enzyme at 3, 10, 18, 25, 30, 35, 40, 45, 50, 55, 60, and 65°C was estimated by ascertaining the residual endo-β-1,4-glucanase activity after completing the hydrolytic reaction that was conducted at pH 4.0 for 10 min. In this case, the enzyme reaction was started by adding 1.0% CMC to the assay mixture after pre-incubation of 1 h at the corresponding reaction temperature in the absence of substrate. The stimulatory or inhibitory effects of metal ions (each 1 mM) and chemical substances (each 5 mM or 0.5%) on the endo-β-1,4-glucanase activity of rGluL were assessed after pre-incubation of the enzyme at 3°C for 10 min in the reaction mixture that included the chemical of concern in the presence of 1.0% CMC. The salt tolerance of rGluL was assessed by reacting the enzyme with CMC for 10 min in the standard assay mixture containing NaCl at concentrations of 0, 0.1, 0.5, 1.0, 2.0, and 4.0 M.

### Identification of the degradation products

The enzymatic hydrolysis of D-cellooligosaccharides (C_2_–C_6_, each 1 mg) and CMC (2 mg) was accomplished by reacting rGluL (10 μg) with the cellulosic substrates in 50 mM sodium citrate buffer (pH 4.0) for 3 h at 45°C, during which the enzyme retained more than 75% of its original endo-β-1,4-glucanase activity. Next, the enzyme reaction was stopped by heating the reaction mixtures at 100°C for 5 min, after which the resulting products formed by the degradation of the cellulosic substrates were identified by liquid chromatography/tandem mass spectrometry (LC-MS/MS) using D-glucose (C_1_) and D-cellooligosaccharides (C_2_–C_6_) as standards. High performance liquid chromatography (HPLC) analysis was conducted employing a Finnigan Surveyor Modular HPLC system (Thermo Electron Co., Waltham, MA, United States) connected with an Asahipak NH2P-50 2D column (5 μm, 2.0 × 150 mm, Showa Denko K.K., Tokyo, Japan). Mobile phase A consisted of water and 0.05% formic acid, whereas mobile phase B contained acetonitrile and methanol at a ratio of 7:3 together with 0.05% formic acid. Degradation products were eluted from the column using the following conditions: a linear gradient of 90–85% B from 0 to 20 min and isocratic elution with 50% B from 20 to 25 min at a flow rate of 0.25 mL/min. Additionally, LC-MS was accomplished by a Finnigan LCQ Advantage MAX ion trap mass spectrometer (Thermo Electron Co.) equipped with an electrospray ionization (ESI) source, as described elsewhere (Kim et al., 2010).

## Results and discussion

### Genetic characterization of the GH8 endo-β-1,4-glucanase gene

**FIGURE 1:**
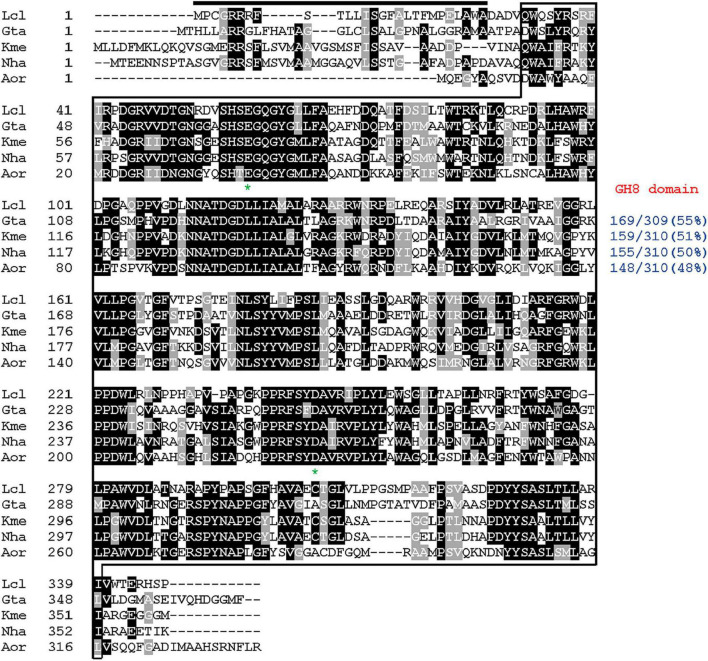
Primary sequence alignment of *Lichenicola cladoniae* PAMC 26568 GH8 endo-β-1,4-glucanase and its structural homologs. Sequences (GenBank accession numbers): Lcl, *L. cladoniae* PAMC 26568 endo-β-1,4-glucanase (ON016586); Gta, *Gluconacetobacter takamatsuzukensis* glycosyl hydrolase family 8 (WP_182950054); Kme, *Komagataeibacter medellinensis* NBRC 3288 endo-β-1,4-glucanase (BAK84916); Aor, *Acetobacter orientalis* endo-β-1,4-glucanase (GAN65284); Nha, *Novacetimonas hansenii* endoglucanase (QOF96800). The identical and similar amino acids are shown by black and gray boxes, respectively. The predicted signal peptide is indicated by a black bar and GH8 domain is outlined by solid line. Highly conserved amino acid residues that play an essential role in biocatalysis are indicated by asterisks.

The 1044-bp GluL gene (GenBank accession number: ON016586) identified by an *in silico* analysis of the complete genome sequence of *L. cladoniae* PAMC 26568 was predicted to encode an extracellular GH8 endo-β-1,4-glucanase comprised of 347 amino acids (Figure 1). Accordingly, the deduced molecular mass and calculated isoelectric point (pI) of the premature GluL were assessed to be 38,630 Da and 7.80, respectively, as analyzed using the Compute pI/MW tool.^2^ In addition, the cleavage site for its signal peptide was predicted to be located between Ala27 and Asp28 in the N-terminal region, as analyzed by the SignalP 6.0 server.^3^ Compared to the premature GluL, the mature form lacking a signal sequence was estimated to be a protein with a deduced molecular mass of 35,576 Da and a calculated pI of 6.73. As displayed in Figure 1, protein BLAST and Pfam analyses revealed that similar to other characterized cold-adapted GH8 functional homologs (Bhat et al., 2013; Chen et al., 2020; de Melo et al., 2021), the premature GluL might be a non-modular endo-β-1,4-glucanase consisting of a single catalytic GH8 domain (from Gln32 to Ile339) without other additional substrate-binding domains such as carbohydrate-binding module and ricin-type β-trefoil lectin domain-like domain.

**FIGURE 2:**
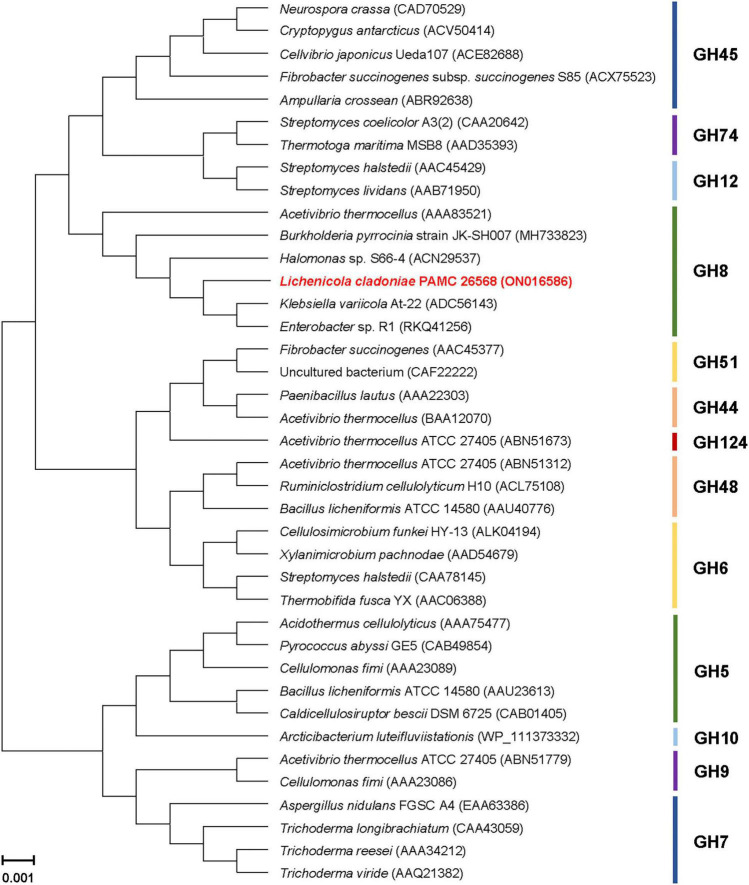
Phylogenetic analysis of *Lichenicola cladoniae* PAMC 26568 GH8 endo-β-1,4-glucanase (GluL) and its closely related functional homologs. Alignment of the amino acid sequences was achieved using ClustalW in the MegAlign program (DNASTAR Inc., Madison, WI, United States). The protein sequence data employed for phylogenetic analysis were retrieved from the GenBank database.

The phylogenetic tree exhibited in Figure 2 clearly revealed that the primary structure of GluL shared a close evolutionary relationship with that of its functional homologs within GH family 8, which constituted inverting endo-β-1,4-glucanase (EC 3.3.1.4), chitosanase (3.2.1.132), licheninase (EC 3.2.1.73), endo-β-1,4-xylanase (EC 3.2.1.8), reducing-end-xylose releasing exo-oligoxylanase (EC 3.2.1.156), and endo-β-1,3-1,4-glucanase/lichenase-laminarinase (EC 3.2.1.6).^4^ Moreover, multiple sequence alignment indicated that the catalytic GH8 domain of GluL was most similar to that of *Gluconacetobacter takamatsuzukensis* glycoside hydrolase family 8 (GenBank accession number: WP_182950054) with a sequence identity of 55%, which had not been functionally characterized but identified only through a genome survey (Figure 1). A protein BLAST survey also revealed that the catalytic GH8 domain of GluL shared a relatively low sequence identity of <53% with that of most uncharacterized structural homologs deposited in the National Center for Biotechnology Information (NCBI) database. For example, the catalytic GH8 domain of GluL was assessed to be 51, 50, and 48% identical to that of *Novacetimonas hansenii* endoglucanase (QOF96800), *Komagataeibacter medellinensis* NBRC 3288 endo-β-1,4-glucanase (BAK84916), and *Acetobacter orientalis* endo-β-1,4-glucanase (GAN65284), respectively, which have not yet been biochemically characterized (Figure 1). In GluL, the two conserved residues related to catalysis, Glu59 (proton donor) and Asp246 (nucleophile/base), were identified in its active site, as observed in other GH8 endo-β-1,4-glucanases (Bhat et al., 2013; Cano-Ramírez et al., 2016; Huang et al., 2021). However, it has also been reported that in the case of a cold-adapted endo-β-1,4-glucanase from *Burkholderia pyrrocinia* JK-SH007, the two Glu83 and Glu271 residues in the active site play a key role in catalysis as the proton donor and the acceptor, respectively (Chen et al., 2020).

### Heterologous expression and purification of (His)_6_-tagged recombinant endo-β-1,4-glucanase

It has been reported that when recombinantly overexpressed in *E. coli* BL21, some carbohydrolases are often produced as inactive inclusion bodies (Kim et al., 2009; Bai et al., 2021a,b), which can be converted to their native state by an on-column refolding protocol (Singh et al., 2015). Of such biocatalysts, a bi-modular GH6 endo-β-1,4-glucanase with a carbohydrate-binding module 2 domain from *Cellulosimicrobium funkei* HY-13 (Kim et al., 2016) and a non-modular GH5 endo-β-1,4-glucanase from a rhizosphere metagenomic library (Wierzbicka-Woś et al., 2019) are examples of enzymes that were normally produced as protein aggregates in an inactive form. However, the rGluL proteins with a (His)_6_-tag at the N-terminus region were intracellularly produced in an active form, similar to other cold-adapted GH8 endo-β-1,4-glucanases (Chen et al., 2020; de Melo et al., 2021). Therefore, based on their solubility, the active (His)_6_-tagged rGluL proteins were simply isolated to electrophoretic homogeneity by basic Ni-NTA affinity chromatography.

**FIGURE 3:**
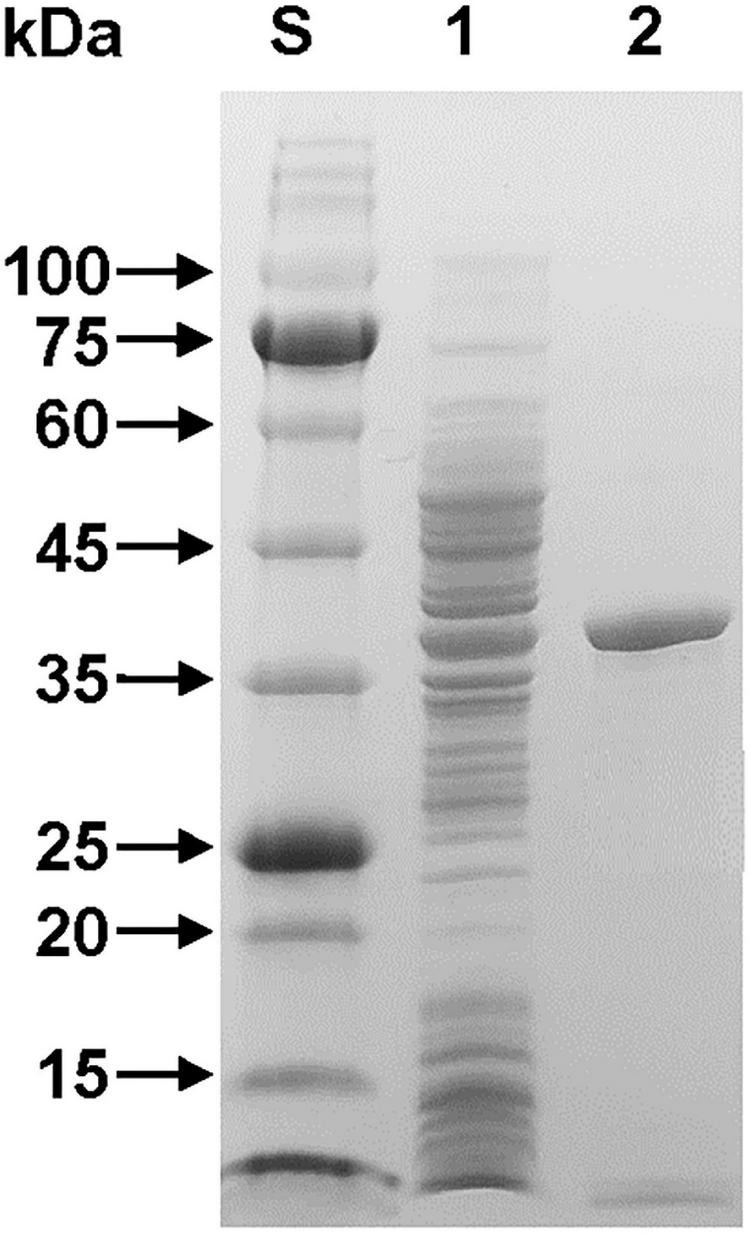
SDS-PAGE of the purified rGluL after affinity chromatography on HisTrap HP. Lane S, standard marker proteins; lane 1, the soluble cell lysate of rGluL-expressing *E. coli* BL21 after IPTG induction; lane 2, purified rGluL.

The relative molecular mass of purified rGluL was estimated to be approximately 38.0 kDa, as analyzed by SDS-PAGE (Figure 3). This value corresponded well to the deduced molecular mass (37,870 Da) of the enzyme that was determined using the Compute pI/MW tool (see text footnote 2). As listed in Table 1, the molecular size (38.0 kDa) of rGluL was assessed to be relatively similar to that of some GH8 endo-β-1,4-glucanases with relative molecular masses between 36.0 and 40.0 kDa. However, its molecular size was found to be much smaller than that of *B. pyrrocinia* JK-SH007 cold-adapted endo-β-1,4-glucanase (60.0 kDa) (Chen et al., 2020) and that of *Bacillus subtilis* B111 endo-β-1,4-glucanase (53.0 kDa) (Huang et al., 2021), which were composed of a single catalytic GH8 domain.

**TABLE 1:** Biocatalytic characteristics of microbial GH8 endo-β-1,4-glucanases.

Source	Enzyme	M*_*r*_* (kDa)	Opt. pH	Opt. temp. (°C)	Specific activity (U mg^–1^)	References
*Lichenicola cladoniae* PAMC 26568	rGluL	38.0	4.0	45	15.1^a^, 8.3^b^	This study
*Xanthomonas citri* subsp. *citri*	*Xac*Cel8	41.5	5.5–7.5	40	16.6^a^, 12.7^b^	de Melo et al., 2021
*Burkholderia pyrrocinia* JK-SH007	BpEG	60.0	6.0	35	11.2^b^	Chen et al., 2020
Cold desert soil in Ladakh	CEL8M	39.0	4.5	28	4.7^b^	Bhat et al., 2013
*Enterobacter* sp. R1	GH8ErCel	38.0	7.0	60	49.8^a^, 4.1^b^	Ontañon et al., 2019
*Bacillus subtilis* B111	celA1805	53.0	6.0	50	12.8^a^, 7.6^b^	Huang et al., 2021
*Bacillus circulans* KSM-N257	Egl-257	43.0	8.5	55	17.7^b^	Hakamada et al., 2002
*Bursaphelenchus xylophilus*	Cen219	40.0	6.0	50	189.6^a^, 107.2^b^	Zhang et al., 2013
*Serratia proteamaculans* CDBB-1961	Cel8A	41.0	7.0	40	0.8^b^	Cano-Ramírez et al., 2016
*Halomonas* sp. S66-4	Cel8H	36.0	5.0	45	1.9^a^, 4.9^b^	Huang et al., 2010

^a^Specific enzyme activity toward barley β-glucan.

^b^Specific enzyme activity toward CMC.

### Biocatalytic characterization of recombinant endo-β-1,4-glucanase

**FIGURE 4:**
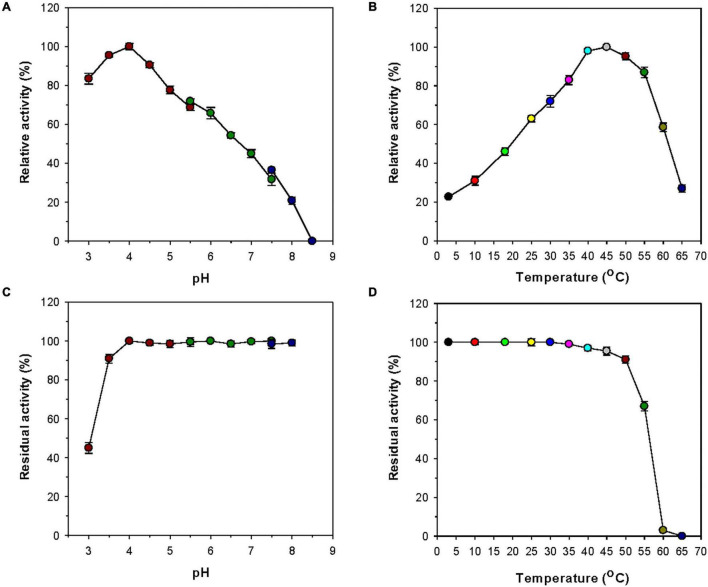
Effects of pH **(A)** and temperature **(B)** on the endo-β-1,4-glucanase activity of rGluL and effects of pH **(C)** and temperature **(D)** on the stability of recombinant endo-β-1,4-glucanase (rGluL). The optimum pH of rGluL was investigated using the following buffers at 50 mM: sodium citrate (pH 3.0–5.5), sodium phosphate (pH 5.5–7.5), and Tris-HCl (pH 7.5–8.5). The optimum temperature of rGluL was determined at various temperatures (3–65°C) in 50 mM sodium citrate buffer (pH 4.0). The pH stability of rGluL was estimated by ascertaining the residual endo-β-1,4-glucanase activity after pre-incubation of the enzyme using the aforementioned buffer systems (50 mM) at 3°C for 1 h. The thermal stability of rGluL was examined by determining the residual endo-β-1,4-glucanase activity after pre-incubation of the enzyme at 3, 10, 18, 25, 30, 35, 40, 45, 50, 55, 60, and 65°C in 50 mM sodium citrate buffer (pH 4.0) for 1 h. The values are mean ± SD of triplicate tests.

Most GH8 endo-β-1,4-glucanases exhibited the highest biocatalytic activity toward CMC or barley β-glucan at a pH value between 6.0 and 7.0 (Table 1). However, rGluL was found to maximally degrade CMC at pH 4.0 and 45°C (Figures 4A,B), indicating that it was an acidic endo-β-1,4-glucanase. The optimum pH of rGluL to decompose CMC was lower than that of a GH8 endo-β-1,4-glucanase (pH 5.0) from *Halomonas* sp. S66-4 (Huang et al., 2010) and that of a cold-active GH8 endo-β-1,4-glucanase (pH 4.5) from cold desert soil in Ladakh (Bhat et al., 2013) identified by functional metagenomics. In contrast to the aforementioned acidic GH8 endo-β-1,4-glucanases, *Bacillus circulans* KSM-N257 GH8 endo-β-1,4-glucanase was an alkaline enzyme that maximally catalyzed the degradation reaction of CMC at pH 8.5 (Hakamada et al., 2002). It is also interesting to note that rGluL displayed more than 80% of its maximum endo-β-1,4-glucanase activity even at pH 3.0, although a gradual decrease in the biocatalytic activity was observed as the pH of the reaction mixture increased in the range of 4.0–8.5. As shown in Figure 4C, rGluL was fairly stable in a pH range from 4.0 to 8.0 because the enzyme maintained over 95% of its residual endo-β-1,4-glucanase activity after pre-incubation of 1 h at those pH values. Conversely, the pH stability of rGluL was significantly reduced by <55% when exposed to pH 3.0 for 1 h in the absence of CMC. The optimum temperature (45°C) of rGluL was higher than that of a cold-active GH8 endo-β-1,4-glucanase (28°C) from Ladakh soil (Bhat et al., 2013), that of a cold-adapted GH8 endo-β-1,4-glucanase (35°C) from *B. pyrrocinia* JK-SH007 (Chen et al., 2020), and that of a cold-adapted GH8 endo-β-1,4-glucanase (40°C) from *X. citri* subsp. *citri* (de Melo et al., 2021). However, similar to the aforementioned cold-adapted GH8 enzymes, rGluL also showed over 60% of its maximum endo-β-1,4-glucanase activity when reacted with CMC at 25°C (Figure 4B). Furthermore, rGluL was capable of easily deconstructing CMC to some extent (approximately 23% of its maximum hydrolytic activity in a cold environment of 3°C), which suggested that the enzyme was a cold-adapted endo-β-1,4-glucanase. It is assumed that the cold adaptation of rGluL might be due to an increase of its structural flexibility for efficient biocatalytic reactions at low temperatures (Parvizpour et al., 2015). rGluL is likely to be relatively thermostable at temperatures below 50°C because it retained over 90% of its residual biocatalytic activity after pre-incubation of 1 h (Figure 4D). In contrast, the enzyme was drastically inactivated when exposed to temperatures exceeding 50°C for the same pre-incubation time in the absence of CMC. The thermal stability of cold-adapted GH8 rGluL was comparable to the thermal instability of a cold-active GH8 endo-β-1,4-glucanase from Ladakh soil (Bhat et al., 2013) and a cold-adapted GH8 endo-β-1,4-glucanase from *B. pyrrocinia* JK-SH007 (Chen et al., 2020), which were observed to lose over 55% of their residual biocatalytic activity at temperatures exceeding 40°C in a pre-incubation period of 1 h.

**FIGURE 5:**
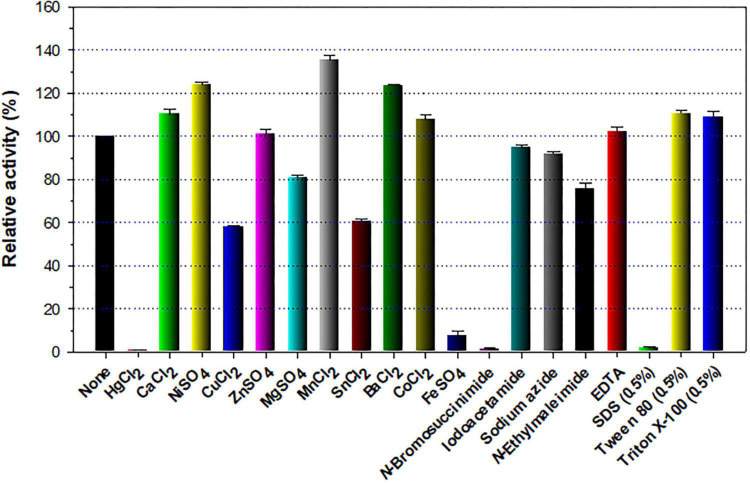
Effects of metal ions (1 mM) and chemical reagents (5 mM) on the endo-β-1,4-glucanase activity of recombinant endo-β-1,4-glucanase (rGluL). The values are mean ± SD of triplicate tests.

**FIGURE 6:**
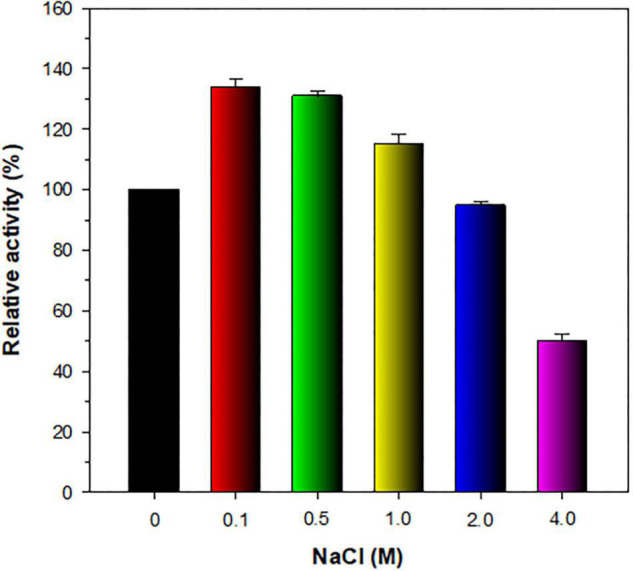
Effect of NaCl on the endo-β-1,4-glucanase activity of recombinant endo-β-1,4-glucanase (rGluL). The salt tolerance of rGluL was evaluated by reacting the enzyme with CMC for 10 min in the assay mixture including NaCl at concentrations of 0, 0.1, 0.5, 1.0, 2.0, and 4.0 M. The values are mean ± SD of triplicate tests.

The stimulatory or inhibitory effect of various divalent cations and chemical reagents on the endo-β-1,4-glucanase activity of rGluL is displayed in Figure 5. When pre-incubated in the presence of 1 mM Ni^2+^, Ba^2+^, or Mn^2+^, the endo-β-1,4-glucanase activity of rGluL was noticeably stimulated by >1.25-fold. In particular, the substantial activation (1.34-fold) of rGluL by 1 mM Mn^2+^ was comparable to the partial inhibition (<80%) of a cold-adapted GH8 endo-β-1,4-glucanase from *X. citri* subsp. *citri* (de Melo et al., 2021) by the same metal ions. Previously, the stimulatory or inhibitory effect of 1 mM Mn^2+^ on the biocatalytic activity of a GH8 endo-β-1,4-glucanase from *Bacillus subtilis* B111 (Huang et al., 2021) and a GH8 endo-β-1,4-glucanase from *Halomonas* sp. S66-4 (Huang et al., 2010) was also shown to be marginal. It is noteworthy that the CMC-decomposing activity of rGluL was partially downregulated by >18% in the presence of 1 mM Mg^2+^, Cu^2+^, Sn^2+^, or *N*-ethylmaleimide, although no enzyme inhibition by 1 mM Zn^2+^ or EDTA was observed. On the other hand, *Halomonas* sp. S66-4 GH8 endo-β-1,4-glucanase was reported to be insensitive to 1 mM Mg^2+^ but partially suppressed by approximately 22% in the presence of 1 mM EDTA (Huang et al., 2010). Moreover, compared to rGluL, *S. proteamaculans* CDBB-1961 GH8 endo-β-1,4-glucanase was completely inactivated when pre-incubated in the presence of 1 mM Zn^2+^, although the stimulatory or inhibitory effect of 1 mM EDTA on the enzyme activity was negligible (Cano-Ramírez et al., 2016). As shown in diverse cellulolytic and hemicellulolytic enzymes (Yin et al., 2010; Kim et al., 2011, 2018; Liu et al., 2021), rGluL was also found to be highly sensitive to tryptophan (Trp) residue-specific modifiers, such as Hg^2+^ and *N*-bromosuccinimide, oxidizing the indole ring of conserved Trp residues in the active site of endo-type GH enzymes that play a key role in the binding of enzyme to substrate (Zolotnitsky et al., 2004). Specifically, it appeared that the enzyme almost completely lost its endo-β-1,4-glucanase activity toward CMC when pre-incubated at 3°C for 10 min in the presence of 1 mM Hg^2+^ or 5 mM *N*-bromosuccinimide without the substrate. Similar observations were also made by a GH8 endo-β-1,4-glucanase from *Halomonas* sp. S66-4 (Huang et al., 2010) and a cold-active GH8 endo-β-1,4-glucanase from Ladakh soil (Bhat et al., 2013) treated with 1 mM Hg^2+^ and/or *N*-bromosuccinimide. However, compared to rGluL, GH8 endo-β-1,4-glucanases from *S. proteamaculans* CDBB-1961 (Cano-Ramírez et al., 2016) and *Bursaphelenchus xylophilus* (Zhang et al., 2013) exhibited approximately 64 and 36% of their maximum CMC-degrading activity in the presence of 5 and 10 mM Hg^2+^ ions, respectively. It is interesting to note that the strong inhibition (>90%) of rGluL by 1 mM Fe^2+^ was very comparable to the powerful stimulation (1.58-fold) of *Halomonas* sp. S66-4 GH8 endo-β-1,4-glucanase (Huang et al., 2010) exerted by the same metal ions. In addition, in the case of a GH8 endo-β-1,4-glucanase from *S. proteamaculans* CDBB-1961 (Cano-Ramírez et al., 2016) and a cold-adapted GH8 endo-β-1,4-glucanase from *X. citri* subsp. *citri* (de Melo et al., 2021), no significant inhibition or stimulation was seen by pre-incubation with 1 mM Fe^2+^. Meanwhile, rGluL was proven to be almost completely inactivated when pre-incubated in the presence of 0.5% SDS, although it was slightly upregulated by approximately 1.1-fold in the presence of a non-ionic detergent (0.5% Tween 80 or 0.5% Triton X-100). The results suggested that SDS was highly toxic to rGluL, as previously exhibited by other characterized GH8 endo-β-1,4-glucanases (Bhat et al., 2013; Zhang et al., 2013). Conversely, the inhibition of *B. subtilis* B111 GH8 endo-β-1,4-glucanase by SDS resulted in an approximately 50% reduction in its original CMC-hydrolyzing activity (Huang et al., 2021). It should also be noted that the endo-β-1,4-glucanase activity of rGluL toward CMC could be remarkably enhanced by >1.3-fold in the presence of NaCl between 0.1 and 0.5 M (Figure 6). Moreover, the suppression of its endo-β-1,4-glucanase activity exerted by 2.0 M NaCl was only observed to be approximately 5%, implying that it was a relatively salt-tolerant enzyme. These findings suggested that the salt tolerance of rGluL was superior to that of *Paenibacillus xylanivorans* A59 GH8 endo-β-1,4-glucanase (Ghio et al., 2020), which showed biocatalytic activity below 50% in the presence of NaCl between 0.5 and 2.0 M.

### Substrate specificity, kinetic parameters, and hydrolytic properties of recombinant endo-β-1,4-glucanase

Different types of true endo-β-1,4-glucanases lacking other additional carbohydrolase activities in addition to bi- and multi-functional endo-β-1,4-glucanases, which belong to GH family 8, have been identified and biochemically characterized to date (Table 1). Bacterial GH8 enzymes from *Enterobacter* sp. R1 (Ontañon et al., 2019), *X. citri* subsp. *citri* (de Melo et al., 2021), *Halomonas* sp. S66-4 (Huang et al., 2010), *B. circulans* KSM-N257 (Hakamada et al., 2002), and *B. pyrrocinia* JK-SH007 (Chen et al., 2020) are examples of true endo-β-1,4-glucanases that preferentially cleaved only β-1,4-glycosidic bond in cellulosic polysaccharides. In contrast, a GH8 endo-β-1,4-glucanase from *B. subtilis* B111 has been recently reported to decompose diverse cellulosic substrates as well as chitosan (Huang et al., 2021). Similarly, bi-functional GH8 endo-β-1,4-glucanases, which were specific to both cellulosic and xylosic polysaccharides, have also been identified from *S. proteamaculans* CDBB-1961 (Cano-Ramírez et al., 2016) and *B. xylophilus* (Zhang et al., 2013). Compared to the aforementioned GH8 endo-β-1,4-glucanases, a cold-active endo-β-1,4-glucanase from Ladakh soil (Bhat et al., 2013) and an endo-β-1,4-glucanase from *P. xylanivorans* A59 (Ghio et al., 2020) were reported to be multi-functional GH8 enzymes capable of deconstructing the polysaccharides with different microstructures, such as CMC, xylan, and chitosan, to some extent. Therefore, the substrate specificity of cold-adapted GH8 rGluL in this study was evaluated using structurally different sugar-based polymeric materials together with *p*NP-sugar derivatives (Table 2). Of the investigated polysaccharides, rGluL was able to preferentially deconstruct amorphous CMC (β-1,4-D-glucan) and barley β-glucan (β-1,3-1,4-D-glucan) to low molecular weight derivatives and with specific activities toward CMC and barley β-glucan estimated at 8.3 and 15.1 U mg^–1^, respectively. However, the enzyme did not exhibit any detectable hydrolytic activity toward Avicel PH-101 (crystalline β-1,4-D-glucan), β-1,3-D-glucan, β-1,4-D-xyloglucan, β-1,4-D-chitosan, β-1,4-D-mannan, β-1,4-D-glucomannan, β-1,4-D-xylan, *p*NP-glucopyranoside, and *p*NP-cellobioside. Based on these findings, it was proposed that like some GH8 endo-β-1,4-glucanases (Huang et al., 2010; Ontañon et al., 2019; Chen et al., 2020; de Melo et al., 2021), the cold-adapted rGluL enzyme is a new type of true GH8 endo-β-1,4-glucanase without other additional glycoside hydrolase activities. It is worth noting that the specific activity (15.1 U mg^–1^) of rGluL for barley β-glucan was approximately 7.9- and 1.2-fold higher than that (1.9 U mg^–1^) of *Halomonas* sp. S66-4 GH8 endo-β-1,4-glucanase (Huang et al., 2010) and that (12.8 U mg^–1^) of *B. subtilis* B111 GH8 endo-β-1,4-glucanase (Huang et al., 2021), respectively. However, the barley β-glucan-degrading activity (15.1 U mg^–1^) of rGluL was assessed to be slightly lower than that (16.6 U mg^–1^) of a cold-adapted GH8 endo-β-1,4-glucanase from *X. citri* subsp. *citri* (de Melo et al., 2021). Likewise, rGluL (8.3 U mg^–1^) was more active on CMC compared to a GH8 endo-β-1,4-glucanase (0.8 U mg^–1^) from *S. proteamaculans* CDBB-1961 (Cano-Ramírez et al., 2016), a GH8 endo-β-1,4-glucanase (4.1 U mg^–1^) from *Enterobacter* sp. R1 (Ontañon et al., 2019), and a cold-active GH8 endo-β-1,4-glucanase (4.7 U mg^–1^) from Ladakh soil (Bhat et al., 2013; Table 1). Conversely, the biocatalytic activity (8.3 U mg^–1^) of rGluL to decompose CMC was estimated to be relatively lower than that (12.7 U mg^–1^) of a cold-adapted GH8 endo-β-1,4-glucanase from *X. citri* subsp. *citri* (de Melo et al., 2021) and that (11.2 U mg^–1^) of a cold-adapted GH8 endo-β-1,4-glucanase from *B. pyrrocinia* JK-SH007 (Chen et al., 2020).

**TABLE 2:** Degradation activity of recombinant endo-β-1,4-glucanase (rGluL) toward different substrates.

Substrate	Main linkage type	Specific activity (U mg^–1^)^a^	Relative activity (%)
Avicel PH-101	β-1,4-D-glucan	ND^b^	–
CMC	β-1,4-D-glucan	8.3 ± 0.1	54.9
Barley β-glucan	β-1,3-1,4-D-glucan	15.1 ± 0.2	100.0
Curdlan	β-1,3-D-glucan	ND	–
Chitosan	β-1,4-D-chitosan	ND	–
Xyloglucan	β-1,4-D-xyloglucan	ND	–
Glucomannan	β-1,4-D-glucomannan	ND	–
Locust bean gum	β-1,4-D-mannan	ND	–
Beechwood xylan	β-1,4-D-xylan	ND	–
*p*NP-glucopyranoside	β-1,4-D-glucan	ND	–
*p*NP-cellobioside	β-1,4-D-glucan	ND	–

^a^Specific activity was obtained from the three repeated experiments.

^b^Not detected.

The kinetic parameters of rGluL for CMC and barley β-glucan in concentration range 0.2–1.2%, which was determined by non-linear regression of the Michaelis-Menten equation, are shown in Table 3. Under the optimal pH (4.0) and temperature (45°C) conditions, rGluL exhibited a *V*_*max*_ value of 27.48 U mg^–1^, a *K*_m_ value of 2.73 mg mL^–1^, and a *k*_cat_ value of 17.34 s^–1^ toward barley β-glucan. In this case, the catalytic efficiency (*k*_cat_/*K*_*m*_: 6.35 mg^–1^ s^–1^ mL) of rGluL toward barley β-glucan was approximately 1.23- and 1.60-fold higher than that (3.98 mg^–1^ s^–1^ mL) of a cold-adapted GH8 endo-β-1,4-glucanase from *X. citri* subsp. *citri* (de Melo et al., 2021) and that (5.16 mg^–1^ s^–1^ mL) of a thermostable GH8 endo-β-1,4-glucanase from *Enterobacter* sp. R1 (Ontañon et al., 2019), respectively, for the same polysaccharide. However, the apparent *k*_cat_/*K*_m_ value of rGluL toward CMC was calculated to be approximately 2.83 mg^–1^ s^–1^ mL, which was lower than its *k*_cat_/*K*_m_ value (6.35 mg^–1^ s^–1^ mL) toward barley β-glucan (Table 3). The results indicated that the kinetic efficiency (2.83 mg^–1^ s^–1^ mL) of rGluL for CMC was slightly higher than that (2.50 mg^–1^ s^–1^ mL) of a cold-active GH8 endo-β-1,4-glucanase from Ladakh soil (Bhat et al., 2013) for the same substrate but was relatively lower than that (6.05 mg^–1^ s^–1^ mL) of a cold-adapted GH8 endo-β-1,4-glucanase from *X. citri* subsp. *citri* (de Melo et al., 2021).

**TABLE 3:** Kinetic parameters of recombinant endo-β-1,4-glucanase (rGluL) determined using carboxymethylcellulose (CMC) and barley β-glucan.

Substrate	*V*_max_ (U mg^–1^)	*K*_m_ (mg mL^–1^)	*k*_cat_ (s^–1^)	*k*_cat_/*K*_m_ (mg^–1^ s^–1^mL)
CMC	14.45	3.22	9.12	2.83
Barley β-glucan	27.48	2.73	17.34	6.35

**FIGURE 7:**
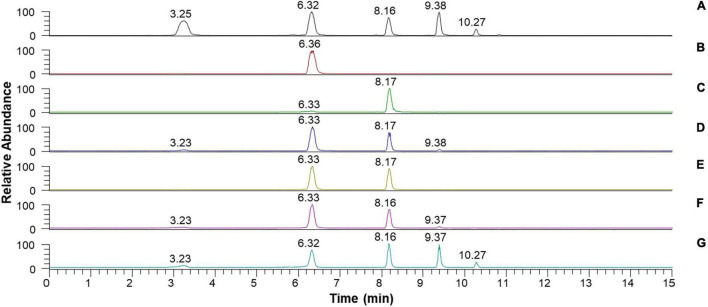
Liquid chromatography (LC) analysis of the degradation products of D-cellooligosaccharides and CMC by recombinant endo-β-1,4-glucanase (rGluL): **(A)** total ion chromatogram of the standards [D-glucose (C_1_: a peak with a retention time of 3.25 min), D-cellobiose (C_2_: a peak with a retention time of 6.32 min), D-cellotriose (C_3_: a peak with a retention time of 8.16 min), D-cellotetraose (C_4_: a peak with a retention time of 9.38 min), and D-cellopentaose (C_5_: a peak with a retention time of 10.27 min)]; **(B)** total ion chromatogram of the degradation products of C_2_; **(C)** total ion chromatogram of the degradation products of C_3_; **(D)** total ion chromatogram of the degradation products of C_4_; **(E)** total ion chromatogram of the degradation products of C_5_; **(F)** total ion chromatogram of the degradation products of C_6_; **(G)** total ion chromatogram of the degradation products of CMC.

Despite the inability of rGluL to cleave C_2_, the enzyme could hydrolyze CMC as well as D-cellooligosaccharides with a degree of polymerization in the range of 3–6, although C_3_ seemed to be only slightly susceptible to the enzyme (Figure 7 and Table 4). Specifically, after the enzymatic hydrolysis of C_3_ for 3 h at pH 4.0 and 45°C, the resultant products in the reaction mixture were identified as C_3_ (93.6%), which was assumed to be unhydrolyzed by rGluL, and a small amount of C_2_ (6.4%). These results implied that the hydrolytic degradation of C_3_ by rGluL was proceeded very slowly without the production of longer D-cellooligosaccharides under the reaction conditions. In contrast to rGluL, five GH8 endo-β-1,4-glucanases from *Enterobacter* sp. R1 (Ontañon et al., 2019), *X. citri* subsp. *citri* (de Melo et al., 2021), *B. pyrrocinia* JK-SH007 (Chen et al., 2020), *P. xylanivorans* A59 (Ghio et al., 2020), and *B. subtilis* B111 (Huang et al., 2021) are known to exhibit no cleavage activity for C_3_, indicative of their lack of exoenzyme activity. Of the evaluated cellulosic substrates, the GluL-mediated biocatalytic degradation of C_5_ resulted in the formation of a mixture consisting of C_2_ (58.2%) and C_3_ (41.8%) without C_1_ and D-cellooligosaccharides with a degree of polymerization of ≥4, suggesting that the enzyme did not have transglycosylation activity. However, the results of HPLC analysis clearly indicated that compared to the inability of *X. citri* subsp. *citri* GH8 endo-β-1,4-glucanase (de Melo et al., 2021) and *B. circulans* KSM-N257 GH8 endo-β-1,4-glucanase (Hakamada et al., 2002) to hydrolyze C_4_, the biocatalytic degradation of C_4_ by rGluL yielded a mixture of C_1_ (3.8%), C_3_ (31.1%), and C_4_ (2.3%) together with C_2_ (62.8%) as the dominant end product. The prominent formation of C_2_ was also identified in a mixture of the degradation products that were formed by the enzymatic hydrolysis of C_5_ and C_6_. The degradation patterns of D-cellooligosaccharides (C_4_-C_6_) were very comparable to those of the same substrates by three GH8 endo-β-1,4-glucanases from *B. pyrrocinia* JK-SH007 (Chen et al., 2020), *X. citri* subsp. *citri* (de Melo et al., 2021), and *Enterobacter* sp. R1 (Ontañon et al., 2019), which resulted in the formation of primarily C_3_ as the end product. Based on these results, rGluL was believed to be a true GH8 endo-β-1,4-glucanase because it could easily degrade CMC, which was generally employed for the assay of endo-β-1,4-glucanase activity in other known GH8 functional homologs (Table 1), to a mixture composed of C_1_ (6.6%), C_2_ (33.9%), C_3_ (30.1%), C_4_ (24.4%), and C_5_ (5.0%) (Figure 7G and Table 4). Conversely, unlike rGluL, a GH8 endo-β-1,4-glucanase from *Halomonas* sp. S66-4 was previously identified as a C_3_ and C_4_-releasing enzyme specific to CMC (Huang et al., 2010). Moreover, a cold-adapted GH8 endo-β-1,4-glucanase from *B. pyrrocinia* JK-SH007 was a biocatalyst releasing exclusively C_1_ from CMC, although it could not hydrolyze C_2_ and C_3_ but catalyzed the hydrolytic degradation of C_4_ and C_5_ that yielded a mixture of C_1_-C_4_ and a mixture of C_1_-C_5_, respectively, as the final degradation products (Chen et al., 2020). Furthermore, no release of C_1_ as the end product was observed after the biocatalytic reaction of CMC with a cold-active GH8 endo-β-1,4-glucanase from Ladakh soil (Bhat et al., 2013), a thermostable GH8 endo-β-1,4-glucanase from *Enterobacter* sp. R1 (Ontañon et al., 2019), and a GH8 endo-β-1,4-glucanase from *X. citri* subsp. *citri* (de Melo et al., 2021). Taken together, the substrate specificity of rGluL and its mode of action on cellulosic substrates elucidated in this study strongly indicated that it was a novel GH8 endo-type β-1,4-D-glucan-degrading enzyme distinct from other characterized GH8 functional homologs (Table 1).

**TABLE 4:** Liquid chromatography analysis of the degradation products of cellulosic materials by recombinant endo-β-1,4-glucanase (rGluL).

	Composition (%)^a^ of products formed by degradation reaction				
Substrate	C_1_	C_2_	C_3_	C_4_	C_5_
C_2_		100.0			
C_3_		6.4	93.6		
C_4_	3.8	62.8	31.1	2.3	
C_5_		58.2	41.8		
C_6_	3.6	59.0	35.0	2.4	
CMC	6.6	33.9	30.1	24.4	5.0

^a^LC area percent.

## Conclusion

The extracellular, non-modular β-1,4-D-glucan-degrading enzyme (GluL) from *L. cladoniae* PAMC 26568, an Antarctic lichen (*C. borealis*)-associated bacterium, is the first microbial GH8 endo-β-1,4-glucanase originated from polar regions that was genetically and functionally characterized. Compared to other previously characterized GH8 functional homologs (Table 1), rGluL is a novel, acidic, and cold-adapted enzyme exhibiting unique characteristics in its primary structure, optimum pH, thermal properties, biocatalytic activities, substrate specificity, and mode of action on cellulosic substrates. Considering its high endo-β-1,4-glucanase activity and thermal properties, the acidic rGluL enzyme can be exploited as a potential biocatalyst in low-temperature textile and food processing industries. From an ecological context, the findings reported in the present study reflect the biological contribution of cellulolytic Antarctic microorganisms, which are responsible for the biological recycling of waste cellulosic materials in the cold environment.

## Data availability statement

The data presented in this study are deposited in the GenBank database repository, accession number: ON016586.

## Author contributions

DK and H-YK designed the study and wrote the manuscript. DK, JK, and SB conducted the biochemical experiments and enzymatic analyses. DK, JK, and JG performed the molecular and phylogenetic analyses. YL provided the bacterial genome sequence. JL conducted the HPLC analysis. DK, YL, D-HS, and H-YK contributed to the data interpretation. All authors contributed to the article and approved the submitted version.

## Conflict of interest

D-HS was employed by Insect Biotech Co. Ltd. The remaining authors declare that the research was conducted in the absence of any commercial or financial relationships that could be construed as a potential conflict of interest.

## Publisher’s note

All claims expressed in this article are solely those of the authors and do not necessarily represent those of their affiliated organizations, or those of the publisher, the editors and the reviewers. Any product that may be evaluated in this article, or claim that may be made by its manufacturer, is not guaranteed or endorsed by the publisher.

## Footnotes

1
http://www.cazy.org/Glycoside-Hydrolases.html

2
https://www.expasy.org/resources/compute-pi-mw

3
https://services.healthtech.dtu.dk/service.php?SignalP-6.0

4
http://www.cazy.org/GH8_characterized.html
